# *Staphylococcus aureus’s* golden-yellow pigment staphyloxanthin: production enhancement, analytical characterization, and biological attributes

**DOI:** 10.1186/s12934-025-02919-2

**Published:** 2026-01-28

**Authors:** Ahmed M. Nosair, Amal M. Abo-Kamar, Lamiaa A. Al-Madboly, Mahmoud H. Farghali, Ahmed A. Abdelaziz

**Affiliations:** Department of Microbiology and Immunology, Faculty of Pharmacy, Tanta University, Tanta, Egypt

**Keywords:** *Staphylococcus aureus*, Staphyloxanthin, Bioproduction, Extraction, Purification, Biological activities

## Abstract

Staphyloxanthin (STX), an apocarotenoid golden yellow pigment produced by *Staphylococcus aureus* isolates, is endowed with potent antioxidant capacity. It plays a crucial role in combating reactive oxygen species (ROS), exhibiting considerable application prospects. This review provides a comprehensive overview of the biosynthetic pathway and various approaches for STX bioproduction in *S. aureus*. Moreover, this review focuses on advancements in biotechnology for enhancing the yield of STX in *S. aureus*, including mutagenesis breeding, metabolic engineering, transcriptional regulation, and the optimization of fermentation conditions. This review addresses the extraction process, purification, and analytical characterization of STX pigment. Additionally, this review highlights the diverse applications of STX in healthcare settings as an antibacterial, antifungal, antiparasitic, antioxidant, anticancer, and DNA damage protection agent. To the best of our knowledge, this is the first review reporting the biotechnological aspects of STX from *S. aureus* as a natural biotechnologically valued product.

## Background

Natural pigments have attracted considerable attention from the industry due to their enhanced biodegradability and safety for human application, which contrasts with synthetic pigments [1]. Microbial pigments present a superior choice owing to their ease of scaling and swift extraction processes, despite the abundance of natural pigments [2]. Bacterial pigments represent a category of secondary metabolites synthesized by microorganisms, serving to enhance their protection and extend their longevity [2]. A multitude of microorganisms produce pigments that fulfill diverse roles, including the regulation of gene expression, the uptake of iron, the facilitation of photosynthesis, and the provision of protection against ultraviolet radiation [3, 4]. The pharmaceutical, food, cosmetic, and textile sectors are driven to utilize bacterial pigments including pyocyanin, indigoidine, rhodopsins, prodigiosin, violacein, pyoverdine, melanin, and carotenoids, owing to their safety, as articulated by Numan et al. [5], Narsing Rao et al. [6], Celedón and Dáz [7], among other researchers. Furthermore, the properties of bacterial pigments, specifically their cytotoxic, antimicrobial, antioxidant, and anticancer capabilities, exhibit a notable correlation with their prospective medicinal applications [8].

Staphyloxanthin (STX) is a golden yellow secondary metabolite pigment and a member of a particular category of apocarotenoid triterpenoid pigments that are developed by *Staphylococcus aureus* [9]. Microbes and plants generate apocarotenoids through the oxidative cleavage of C40 isoprenoids. The synthesis of apocarotenoids is executed through both enzymatic and non-enzymatic processes, with a substantial dependence on carotenoid cleavage dioxygenases (CCDs). The increasing attention to apocarotenoids as natural food colorants has been driven by the escalating negative consequences of synthetic colorants [10]. The cellular concentration of carotenoids and apocarotenoids is remarkably low, despite their extensive distribution in nature [11]. By employing well-established fermentation strategies and specific microbial strains, microbial biotechnology has enhanced the efficiency and cost-effectiveness of microbial production of carotenoids and apocarotenoids [12]. Industrially, apocarotenoids and carotenoids are highly valued for their substantial utility, which is ascribed to their health-promoting effects and anticancer qualities [11, 12]. The biological potential of STX is primarily attributed to its role in generating reactive oxygen species (ROS), which results in oxidative stress and the inhibition of ion-membrane interactions and active solute transport, consequently affecting cellular permeability [9]. First, we conduct a comprehensive analysis of the STX biosynthesis pathway in *S. aureus*. Secondly, this review compiles pertinent information regarding the production of STX pigment, its extraction, purification, and characterization methods, and emphasizes their potential biotechnological applications.

### Biosynthetic pathway of staphyloxanthin

The polyene π-electrons’ profuse delocalization is the most critical structural characteristic of carotenoids, as it allows them to absorb visible light, thereby granting them an intense color from yellow to red [13]. As of 1972, Marshall and Rodwell designated the primary orange pigment of *S. aureus* as STX. The chemical structures of 17 intermediary compounds were identified by Marshall and Wilmoth after extracting the pigments from *S. aureus* S41 with methanol [14]. A C30 structure has been identified in all triterpenoid carotenoids, contrasting with the conventional C40 carotenoid structure prevalent in most organisms. STX, characterized as an α-D-glucopyranosyl 1-O-(4,4ʹ-diaponeurosporene-4-oate)6-O-(12-methyltetradecanoate), served as the principal pigment [14]. However, using NMR spectroscopy in 2005, STX was shown to be β-D-glucopyranosyl-1-O-(4,4ʹ-diaponeurosporen-4-oate)-6-O-(12-methyltetradecanoate) [15]. This structure was created by esterifying glucose with a C15 fatty acid at the C6” position and a triterpenoid carotenoid carboxylic acid at the C1” position [15].

**Fig. 1:**
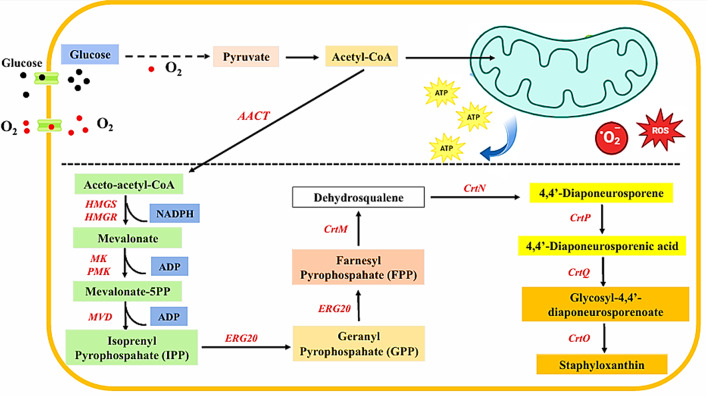
The biosynthetic pathways of staphyloxanthin in *S. aureus*. The following are the enzymes: (1) Mevalonate pathway: MVD, mevalonate diphosphodecarboxylase; PMK, phosphomevalonate kinase; MK, mevalonate kinase; HMGS, hydroxymethylglutaryl-CoA synthase; HMGR, 3-hydroxy-3-methylglutaryl-CoA reductase; AACT, acetyl-CoA acetyltransferase. (2) Isoprenoid pathway: ERG20, geranyl pyrophosphate synthase. (3) Carotenoids pathway: CrtM, dehydrosqualene synthase; CrtN, dehydrosqualene desaturase; CrtP, diaponeurosporene oxidase; CrtQ, glycosyltransferase; CrtO, acyltransferase. The solid arrows denote a reaction occurring in a single step. The dashed arrows signify multistep reactions

The proposed biosynthetic pathway of STX transpires primarily through three distinct stages: the mevalonate pathway, the isoprenoid biosynthetic pathway and the STX biosynthetic pathway [16, 17] (Fig. 1). The transformation of carbon substrates (glucose, mannitol, etc.) into pyruvate is the preliminary phase of the isoprenoid biosynthetic pathway [18]. Subsequently, the pyruvate dehydrogenase complex converts pyruvate to acetyl-CoA via the glycolytic pathway, which is then oxidized to facilitate the ensuing process. Furthermore, the tricarboxylic acid cycle (TCA) produces significant amounts of nicotinamide adenine dinucleotide phosphate hydrogen (NADPH) and adenosine triphosphate (ATP) as a fraction of acetyl-CoA is introduced. These compounds supply energy and reducing potential for the subsequent transformation of substances. The mevalonate, isoprene, and carotenoid synthesis pathways are utilized in the biosynthesis of STX in *S. aureus* [16]. The initial step in the synthesis of acetyl-acetyl-CoA from glycolysis involves the condensation of two acetyl-CoA molecules by acetyl-CoA acetyltransferase (AACT). Subsequently, hydroxymethylglutaryl-CoA synthase (HMGS) further condenses acetyl-acetyl-CoA molecules to produce 3-hydroxy-3-methylglutaryl-CoA (HMG-CoA). Mevalonate is produced as a result of the reduction phase, which is carried out by 3-hydroxy-3-methylglutaryl-CoA reductase (HMGR). At this point, mevalonate diphosphodecarboxylase (MVD), phosphomevalonate kinase (PMK), and mevalonate kinase (MK)mediate a three-step reaction that generates isopentenyl pyrophosphate (IPP).

In the endogenous synthesis of gibberellins, sterols, sesquiterpenes, monoterpenes, carotenoids, and other compounds, IPP serves as a crucial component and prevalent precursor [18]. The IPP is isomerized by bifunctional IPP isomerase (idi) and subsequently condensed to geranyl pyrophosphate (GPP) to dimethylallyl pyrophosphate (DMAPP) through the mevalonate (MVA) pathway by geranyl pyrophosphate synthase (ERG20) in the isoprene biosynthesis pathway. Subsequently, farnesyl diphosphate synthase (ERG20) converts it to farnesyl pyrophosphate (FPP). Subsequently, it engages in the carotenoid synthesis pathway, wherein FPP is catalyzed by dehydrosqualene synthase (CrtM), resulting in the production of dehydrosqualene. Dehydrosqualene is subsequently converted to 4,4’-Diaponeurosporene by the continuous action of dehydrosqualene desaturase (CrtN). 4.4’-Diaponeurosporenic acid is subsequently synthesized from 4,4’-Diaponeurosporene by diaponeurosporene oxidase (CrtP). The glycosyltransferase (CrtQ) action subsequently generated the colored intermediate Glycosyl-4,4’-diaponeurosporenoate. Ultimately, the golden yellow STX pigment was generated through the action of acyltransferase (CrtO) on Glycosyl-4,4’-diaponeurosporenoate [16, 17].

### Regulatory genes affecting staphyloxanthin biosynthesis

In 1981, It was reported that the biosynthetic pathway of the triterpenoid carotenoids of S. aureus is presumed [14]. The cloning of the genes and the analysis of the functions of CrtN and CrtM enzymes involved in STX biosynthesis have verified this pathway, as demonstrated by Wieland et al. [19] In addition, the findings suggested that yellow pigments were exclusively detected in clones that contained intact *crtN* and *crtM* [19]. In 2005, protein sequence similarity comparisons and products analysis of *crt* mutants were employed to analyze the complete STX biosynthesis operon *crt*OPQMN [15]. A σB-dependent promoter was situated upstream of *crtO*, and the operon *crt*OPQMN contained the five genes involved in STX production [15]. Xue et al. conducted a comprehensive analysis of the *crt* gene cluster across 58 completed genomes of *S. aureus*, as documented in the NCBI database, to ascertain the ubiquity of the *crt*OPQMN within this species [17]. The alignment of crtOPQMN protein sequences was conducted across 58 distinct strains of *S. aureus*. The *crt* gene cluster was identified in 58 *S. aureus*, exhibiting a comparable gene arrangement as indicated by the study. The results indicated that the *crt* genes can predominantly be categorized into nine principal groups [17].

**Fig. 2:**
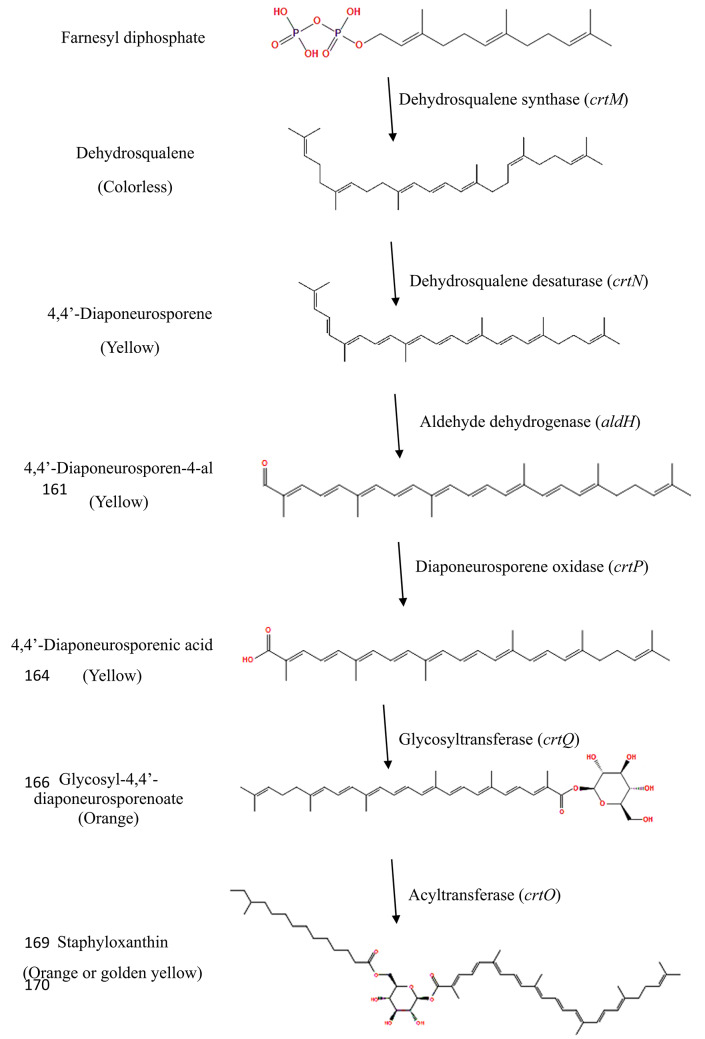
Schematic representation of intermediary structures involved in staphyloxanthin biosynthesis with corresponding enzymatic reactions and their regulatory genes (in parentheses)

In 2012, the complete STX biosynthetic pathway’s functional expression in *Escherichia coli* was described by Hyeuk Kim et al. [20], who also reported the absence of a sixth enzyme, 4,4-diaponeurosporen-aldehyde dehydrogenase (AldH), in the STX biosynthetic pathway. The five known pathway enzymes were expressed in *E. coli* through the wild-type operon (crtOPQMN) and artificial synthetic operons. As a result, STX and various carotenoid aldehyde intermediates remained unconverted, leading to the accumulation of 4,4-diaponeurosporen-4 and other intermediary compounds. An *aldH* gene was identified in the non-STX-producing *Staphylococcus carnosus* genome and the STX-producing *S. aureus* genome, which is located 670 kilobase pairs from the known STX gene cluster. The missing oxidation reaction in *E. coli* was catalyzed by these two putative enzymes, which are responsible for the production of 4,4-diaponeurosporenoic acid. The role of AldH in in STX biosynthesis was confirmed by the accumulation of 4,4-diaponeurosporen-4-al and the cessation of STX biosynthesis in *S. aureus* following the deletion of the *aldH* gene [20]. The schematic representation in Fig. 2 elucidates the enzymatic reactions and the regulatory genes implicated in the biosynthesis of STX.

]

A sigma B (σB) dependent promoter is located downstream of *crtN* and upstream of *crtO*, regulating the operon *crtOPQMN* [21]. A series of Rsb proteins encoded by *rsb* genes (*rsbUVWsigB*) regulate the activity of SigB [22, 23]. The *rsbUVWsigB* system and *crtOPQMN* operon are fundamental for the pigmentation of *S. aureus*, as demonstrated by other studies [24, 25]. In addition to these genes, AirR, an aeriation-sensing response regulator [26], and CspA, a cold-shock protein [27], have been documented to exert a beneficial influence on both *sigB* and pigmentation [26, 27]. The expression of *crtMN* and *sigB* is reduced as a result of the loss of CspA [27]. Furthermore, the expression of *sigB* and the pigmentation of bacterial cells may be influenced by mutations and/or altered activities of certain regulators, such as the staphylococcal ClpP protease, arginine regulator (*argR*), accessory gene regulator (*agrA*), and accessory regulator (*sarA*) [24, 28].

All organisms possess the Heat shock protein family, which encompasses the molecular chaperones referred to as DnaK proteins [29]. DnaK in *S. aureus* is crucial for safeguarding the microorganism against oxidative stress, thermal injury, and antibiotic exposure. DnaK significantly influences autolysis, pigmentation, and in vivo animal survival [30, 31]. *S. aureus dnaK* mutants can produce pigment; however, their colonies exhibit a pale yellow-orange hue attributed to diminished carotenoid synthesis. Furthermore, the mutants exhibited a decreased in vivo survival rate and increased susceptibility to oxidative stress [32]. A multifunctional regulator in bacteria, the Hfq protein was considered to be an RNA chaperone and a host factor for the RNA phage Qβ [33]. The mRNA level of *crtM* could be increased by deleting the *hfq* gene in *S. aureus* 8325-4, a SigB deficiency mutant that is non-pigmented [34]. This implies that *hfq* negatively regulates *crtM* expression in the *sigB*-deficient mutant. However, the yellow carotenoid pigmentation could be increased [34, 35].

It was demonstrated that the inactivation of tricarboxylic acid cycle genes (SAV2365, *citG*, and *citZ*) increased farnesyl diphosphate production by increasing the flux of acetyl-CoA to the mevalonate pathway, which led to increased pigmentation [36]. Additionally, there was no increase in the expression of *sigB* or *crtM* with the acetyl-CoA transfer to the mevalonate pathway. In a similar vein, the inhibition of oxidative phosphorylation genes (*ctaA* and *qoxB*) increases bacterial pigmentation. An increase in the level of *crtM* mRNA was observed in the Δ*ctaA* mutant of *S. aureus*. In contrast, the *qoxB* mutant did not exhibit any substantial changes in the levels of *crtM* and *sigB* mRNA. In addition, the augmentation of *sigB* expression was the explanation for the increased bacterial pigmentation observed in purine biosynthetic (purA, purD, purH, or purN) mutants [17, 25, 36]. An interruption in the production of acetyl-CoA from pyruvate, a substrate for both fatty acid and STX biosynthesis, results from a mutation in pyruvate dehydrogenase (*pdh*). This, in turn, results in a decrease in the biosynthesis of pigment. On the flip side, enhanced pigments biosynthesis was demonstrated by the branched-chain α-keto acid dehydrogenase (*bkd*) mutant. The reason for this is that branched chain fatty acid (BCFA) biosynthesis does not consume acetyl-CoA, which is available for STX biosynthesis [17, 25, 37].

### Biotechnological production of staphyloxanthin

The principal biotechnological approaches aimed at augmenting the STX yield of *S. aureus* encompass mutagenesis breeding, genetic modification, and the optimization of fermentation processes. Various methodologies such as mutagenesis and optimization have been utilized to evaluate a multitude of high-yield strains. The application of specific advanced biotechnologies can enhance our understanding of the metabolic pathways and regulatory mechanisms associated with STX, while also facilitating the creation of innovative alternatives for its industrial production.

### Mutagenesis

The initial strategy for altering natural organisms to serve human interests was undertaken by breeders, who commenced the recombination of diverse genetic materials to develop new strains that embodied the traits of both ancestral organisms [38]. Mutagenesis is the most common direct method for increasing the content of the target product in *S. aureus*, which is responsible for STX production. The strategies employed include exposing *S. aureus* cells to phage transduction and transposon mutagenesis [39]. The Nebraska transposon mutant library (NTML) was generated through the map transpositions of *bursa aurealis* from the delivery plasmid pBursa into the genome of *S. aureus* JE2 [40]. Fey and colleagues discovered that the presence of transposon insertions in the first third of the length of the afflicted gene (*crt*) resulted in the selection of over two-thirds (68%) of the NTML transposon mutant clones with augmented STX pigmentation [40]. Pigment production differences were also observed in other mutants that were involved in earlier enzymes in the biosynthetic pathway of STX. For instance, the SAUSA300_1470 gene, which encodes geranyltranstransferase, is involved in the biosynthesis of farnesyl diphosphate, a critical precursor for the biosynthesis of heptaprenyl diphosphate and carotenoid pigments. It is intriguing that a mutation that affects SAUSA300_1359, a gene that encodes polyprenyl synthetase, the enzyme responsible for converting farnesyl diphosphate to heptaprenyl diphosphate, leads to a significant increase in the production of carotenoid pigments [40].

**Fig. 3:**
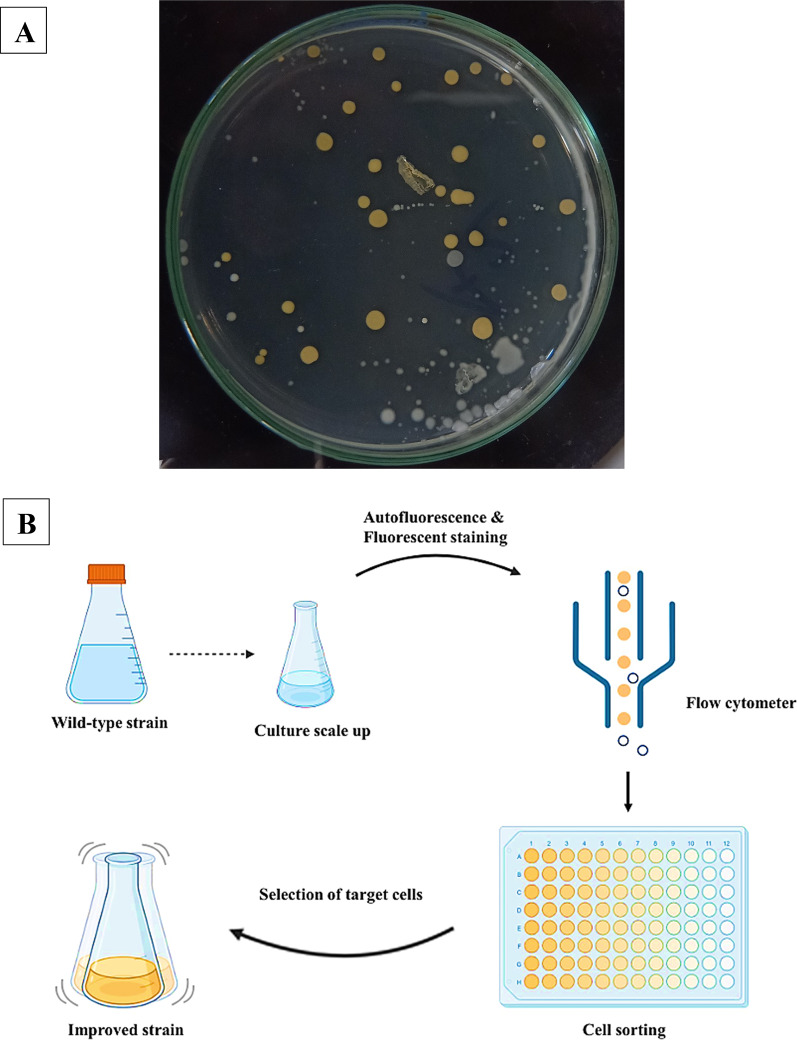
Strategies for random mutagenesis selection with augmented pigmentation.** A** Visual screening and selecting the mutant cells with the highly-pigmented colony (orange color). ** B** Schematic diagram illustrating the steps of flow cytometry screening, which include culturing of the mutant cells, sorting cells based on their autofluorescence of pigments, and then selecting the desired cells with higher fluorescence by physical separation

A phage-80α transduction was employed to introduce a mutation into the *crt* genes of *S. aureus* strain SH1000 [21]. The *crt* mutant was genetically complemented by cloning a DNA fragment into the shuttle plasmid pCU1 and then transferring it to the *crt* mutant strain. This fragment contained 436 nucleotides upstream of the *crtO* gene, the complete set of genes that encode the STX pathway (*crtOPQMN*), and 337 nucleotides downstream of the *crtN* termination codon. In comparison to the wild-type SH1000, the *crt*-complemented mutant produced dark pigmentation of STX, as observed by Pannu et al. [21]. A valuable tool for the production of STX or carotenoid precursors, these mutants are obtained through random mutagenesis. It is imperative to employ efficient selection methods in order to identify the most appropriate mutants, as these methods produce a significantly large number of mutant colonies [41]. Visually screening for STX excess producers is the most common and straightforward method, as it is dependent on changes in color intensity, which are related to their orange color (Fig. 3). Nonetheless, the intricacies of the process are exacerbated by the color saturation and background, which stem from the interference of other intermediates [41]. To discern high-yield pigment producers, alternative screening methodologies, such as flow cytometry, the autofluorescence of pigments (Fig. 3), were employed [41]. However, the efficacy of mutagenesis and the subsequent genetic stability present significant challenges in the selection process for high-yielding strains [18].

.

### Metabolic engineering

Metabolic engineering involves the application of recombinant DNA technology to alter specific metabolic reactions or to introduce novel ones, aiming to enhance biological traits [42]. The engineering of non-carotenogenic microorganisms for the purpose of carotenoid production serves as a valuable asset in today’s context [43]. The potential to improve public health through biotechnology is substantial, particularly by metabolically engineering microorganisms to generate increased quantities of essential molecules [44]. The expression of cloned genes constitutes a pivotal process in the realms of molecular biology and biotechnology, enabling researchers to produce and scrutinize particular products of interest [45]. The initial stage in biotechnological processes to enhance the production of metabolites is the selection of an appropriate microorganism [46]. Microorganisms engineered to generate carotenoids, such as *E. coli*, provide numerous benefits, including the ease of genetic modification with existing host-vector systems [47]. Hyeuk-Kim and colleagues attempted to produce STX in *E. coli* through the cloning and expression of the encoded genes. The biosynthetic process was thoroughly examined, and *E. coli* was modified to produce STX [20]. The authors employed the cloning and expression of pBBR1MCS-2-derived vectors, which were conducted using *E. coli* XL1-Blue [48]. The constitutive expression vector pUCM was used to clone the genes encoding *crtO*, *crtQ*, *crtP*, *crtN*, and *crtM* from *S. aureus* KCTC 1928. From the genomic DNA of *S. aureus* KCTC1928, four genes were amplified and cloned into the pUCM vector; these genes encode an aldehyde dehydrogenase family protein, aldehyde dehydrogenase homolog (*aldA*), glycinebetaine aldehyde dehydrogenase (*gbsA*), and AldH (*aldH*). Finally, complete STX Pathway was expressed in engineered *E. coli* with boosted production of STX pigment [20].

### Transcriptional regulation of STX production

The production of STX is a complex process, and despite the extensive research that has been conducted, there is still a lack of understanding regarding the regulation of transcription factors by *S. aureus*. By improving our understanding of the metabolic process and regulatory mechanism of STX, the production efficiency of STX can be substantially improved through the investigation of STX-related transcription factors produced by *S. aureus*.

The global stress response regulator SigB modulates the expression of the golden yellow pigment. *S. aureus* has the capability to increase the expression of various resistance genes, including those linked to oxidative stress resistance, as demonstrated by the notable STX pigment, which is attributed to the presence of SigB [49]. In order to identify SigB’s protective function against radiation damage, a recent study looked into *S. aureus*’ radiation survival. Different forms of radiation were applied to mutant strain cells of *S. aureus* that lacked the STX pigment (*crt* enzymes-deficient) [50]. Compared to the wild-type strain, the *crt*-mutant cells were shown to be three times more vulnerable to UV light due to deficiency of STX accumulation. RNA polymerases are recruited to the promoter by SigB, which triggers the transcription of the STX pathway genes [21]. Consequently, STX pigmentation is lacking in *S. aureus* sigB mutants. Since proline substituted for glutamine-225 in SigB produced non-pigmented *S. aureus* colonies, it was concluded that this amino acid is crucial for controlling the production of STX. Therefore, overexpression of sigB enhances the production of golden yellow STX pigment in *S. aureus* [21].

The production of STX is regulated and facilitated by two-component signal transduction systems (TCSs), which play a crucial role in staphylococcal metabolism [51]. The AirSR TCS, which is a global regulator that regulates the pathways for lactose catabolism, nitrate respiration, and STX biosynthesis, is capable of sensing oxygen and utilizing redox signaling [52]. It was noted that the colony pigmentation was enhanced by the overexpression of the *airR* response regulator in the genetic analysis of the numerous AirSR mutant strains [52]. The *crtOPQMN* operon is directly transcriptionally activated by the AirSR TCS, which also modifies the synthesis of STX carotenoid. According to Hall et al. [26], *S. aureus* colonies’ golden pigmentation is enhanced by excessive AirR production. The researchers developed isopropyl–D-thiogalactopyranoside (IPTG)-inducible WCUH29 Δ*airR*: :P*spac*-*air*R and WCUH29 Δ*air*S: :Pspac-*air*S mutant *S. aureus* strains. Subsequently, semiquantitative reverse transcription (RT)-PCR demonstrated that airS expression was almost threefold greater than the endogenous airS expression when the P*spac* promoter inducer IPTG was not present. This was probably due to the activity of the “leaky” Pspac promoter. Therefore, The production of STX correlates with the level of AirR produced by *S. aureus* WCUH29 [26].

By offering protection from oxidative stress caused by the host immune system and environmental stress, STX promotes *S. aureus* survival [53]. The hypothesis was that oxidative stress exposure, such as H₂O₂ and UV radiation, would induce an upregulation of stress response genes, particularly those that are responsible for mitigating oxidative stress [53]. The production of STX was substantially increased as a result of the increased expression of genes in the crt operon under H2O2 exposure [54]. A well-characterized phosphatase regulator of the sigB operon, encoded by the *rsbU* gene, dephosphorylates RsbV in response to environmental stress, preventing RsbW from inhibiting sigB. The *crt* biosynthesis operon for STX is subsequently positively regulated by the transcription factor sigB. The *rsbU* mutation is most likely the cause of the decreased STX production in *S. aureus* isolates, in addition to the fact that this non-synonymous SNP correlates to an amino acid alteration in the protein’s catalytic domain [54]. According to other research, the generation of STX required *rsbU*. However, this variant involves a protein sequence alteration that might affect, but not necessarily eliminate, the regulatory function of RsbU. This prompted us to look into the transcriptome variations between *S. aureus* isolates with high and low STX levels as well as the transcriptional responses of the strains to oxidative stress [55].

### Optimization of fermentation conditions

**Fig. 4:**
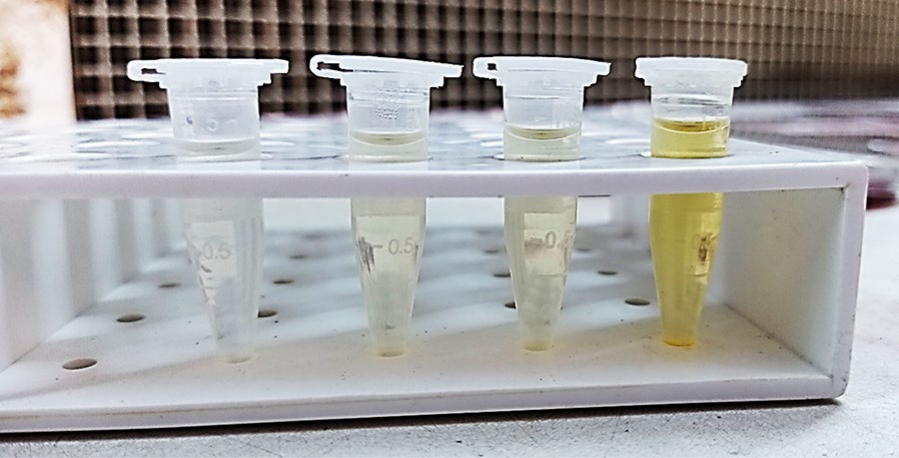
The influence of incubation media composition on STX production using the same *S. aureus* strain. Orange color indicates the higher pigmentation of STX

**Fig. 5:**
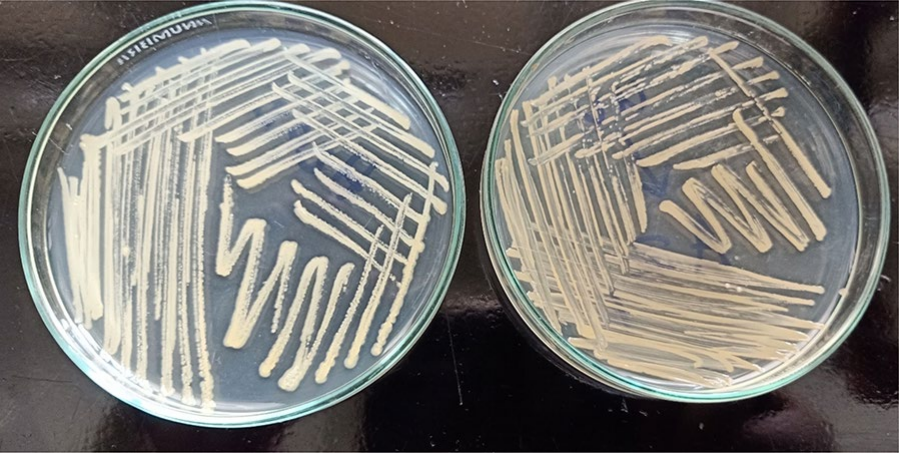
Variable STX pigmentation of the different *S. aureus* isolates screened on nutrient agar plates

The industrial production of microbial pigments must prioritize low-cost processes, high yields, and environmental sustainability. Numerous culture variables, including feeding pattern, pH, dissolved oxygen, and medium composition, also affect the biosynthesis pathway of STX in *S. aureus*. Therefore, by modifying the fermentation conditions, STX biosynthesis can be controlled macroscopically. The amount of STX pigmentation varies among different *S. aureus* stains, as illustrated in Figs. 4 and 5. The most prevalent method for the production of carotenoids is submerged fermentation, which employs a variety of optimization strategies, such as one-factor-at-a-time (OFAT) and multifactorial optimization [18]. The determination of the fermentation media represents the initial crucial step in selecting the most suitable basal media for investigating the influence of various nutritional components and physical conditions [56]. These bioprocess production-related factors have the potential to impact yields and operating expenses. Regardless of the method by which metabolites are produced, each organism has different key physicochemical and nutritional process factors [57]. The process variables needed for metabolite formation in a particular bioprocess may be regulated by the physiological and metabolic traits of the organism involved. The cellular growth and accumulation of metabolic products are both influenced by the medium’s composition. Consequently, the nutritional requirements assessment and evaluation are essential components of bioprocess development [58].

The OFAT optimization of STX production indicated that pigmentation was regulated by the incubation period and the specific type of medium. On low-nutrient media, such as peptone water and glucose broth, the pigmentation was less apparent, while it was more prominent on nutrient-rich medium. These results were consistent with other research that demonstrated that the production of bacterial pigments was increased by increased nitrogen and carbon sources in the fermentation media [59]. The carbon source is the parameter that has been the subject of the most extensive research in order to influence carotenogenesis. The metabolism of microorganisms is contingent upon the kind of carbon supply present in the media. The glycolytic pathway metabolizes the fermentable sugars by facilitating their entry into the citric acid cycle through acetyl-CoA oxidation. Consequently, the mevlonate and isoprenoid pathway are stimulated, thereby enhancing carotegenesis. It has been noted by numerous authors that the synthesis of carotenoids is stimulated by carbon sources, such as fermentable sugars [60, 61]. A recent study examined the effect of incorporating various carbon sources into fermentation media on STX production. The findings indicated that the incorporation of mannitol sugar as an optimal carbon source at a concentration of 0.5 g/L enhanced the growth of S. aureus and promoted STX production [9].

Bacterial species’ pigmentation can be influenced by environmental stress factors, including temperature, pH, and salt concentration. Remarkably, a previous study on *Rhodotorula slooffiae*’s optimum carotenoid pigment production at 37 °C revealed a progressive decrease in pigmentation as the temperature rose over 40 °C [62]. Given that temperature affects both bacterial metabolism and cellular viability, this could be explained by the fact that high temperatures inhibit bacterial growth. The enzyme concentration involved in carotenoid production is regulated by the temperature effect, which in turn regulates the carotenoid levels in microorganisms. The findings indicated that the optimum was achieved at 37 °C, which established an immediate connection between pigment synthesis and biomass and a necessity for growth in order to produce pigments [63]. It was discerned that the optimum stress variables for STX generation from *S. aureus* were a pH of 7 and a temperature of 37 °C [9]. Moreover, an environmental stressor is also considered the modification of the microstructure of the growth medium, which affects the interaction between a variety of environmental variables, including temperature, pH, and water activity, and microbial growth [64]. Immobilized cells have a slower growth rate and different metabolic activities than planktonic cells, as several studies have shown [65, 66]. The rate of growth of *Bacillus cereus* cells cultivated in broth and those immobilized on gelatin gel was shown to differ significantly, according to another study [67]. Similarly, studies have demonstrated that *Aspergillus carbonarious* cultivated in gel growth medium not only grows more slowly (as determined by an increase in biomass), but it also generates less ochratoxin A compared to culture in liquid media [68].

Another factor to consider in carotenoid production is the duration of incubation, which influences cell growth and metabolite production, altering the carotenogenesis and other biosynthesis processes. The impact of incubation is contingent upon the quantity of nutrients and the microorganism. As per an earlier publication, the production of STX pigmentation achieved its lowest point at 96 h and maximized at 72 h during the experimental settings [9]. The duration of incubation may be prolonged to enhance pigmentation; however, beyond 72 h, there is a decline in pigment synthesis. This phenomenon occurs due to the degradation of cells, which results in a diminished capacity to assimilate nutrients. The initiation of the organism’s mortality phase, coupled with the exhaustion of essential nutrients, may serve as contributing elements to the results observed [56]. Additionally, another potential reason for the decrease in pigment production could be the breakdown of the pigment brought on by interactions with different medium components [56, 62, 69].

The incorporation of minerals, which offer critical nutrition and energy sources, as well as physical and chemical protections, improves the developmental capacities of a microbial community [70]. The activity of enzymes is dependent on the presence of micronutrients. They serve as cofactors or prosthetic groups for critical metabolic enzymes in numerous instances, thereby contributing to metabolic regulation [71]. Incorporation of ZnCl_2_ as micronutrient in the medium composition showed a positive impact on STX production [9]. According to earlier studies, the inclusion of mineral and vitamin sources in the fermentation media increased the formation of metabolic products [56, 71]. It has also been shown that metal ions and salts (Ba, Fe, Mg, Ca, Zn, and Co) stimulate *Rhodotorula glutinis* to produce carotenoids. The activation of particular carotenogenic enzymes is responsible for the action of the trace elements indicated [72].

Carotenogenesis is enhanced by the consideration of light, which is a critical factor in the production of microbial carotenoids. The photoprotective mechanism of carotenogenesis is necessary for microorganisms to defend themselves from the light that causes damage. Carotenoid production is positively influenced by white light, and the concentration of carotenoid is contingent upon the microorganism. Additionally, the enhanced activity of enzymes involved in carotenoid biosynthesis is linked to the production of carotenoid. The relationship between carotenoids and cell UV-light resistance in *S. aureus* strains was investigated by previous study reported that hyper-pigmented strains exhibited improved survival. The researchers also suggested that the survival of *S. aureus* in UV-light is enhanced by an increase in STX production [73].

### Multifactorial optimization of fermentation variables using response surface methodology

The mathematical and statistical analysis of multivariate data obtained through response surface methodology (RSM) is a critical component of the optimization and enhancement of metabolite production. The response surface method, a software-based statistical design, is the most widely recognized method for the production of biological molecules, particularly in submerged fermentation. The most significant objective of bioprocess production is to enhance system performance and increase process efficiency without increasing cost and time. The fundamental objective of “multifactorial optimization” is to identify the optimal combination of factors that deliver the most effective response for a system. For the purpose of making statistical predictions, regression and statistical modeling (RSM) involve fitting a polynomial model to data in such a way that it accurately depicts the behavior of a data collection. In addition, the settings of the design factors are selected in order to improve or maximize the performance or responsiveness. It is a general-purpose technique that optimizes the anticipated value of a stochastic response by providing a combination of design of experiments, regression analysis, and optimization methods. There are a number of mathematical models and experimental designs that are utilized in the biotechnological production of pigments, including the Box-Behnken Design, the Factorial Design, and the Central Composite Design (CCD).

**Fig. 6:**
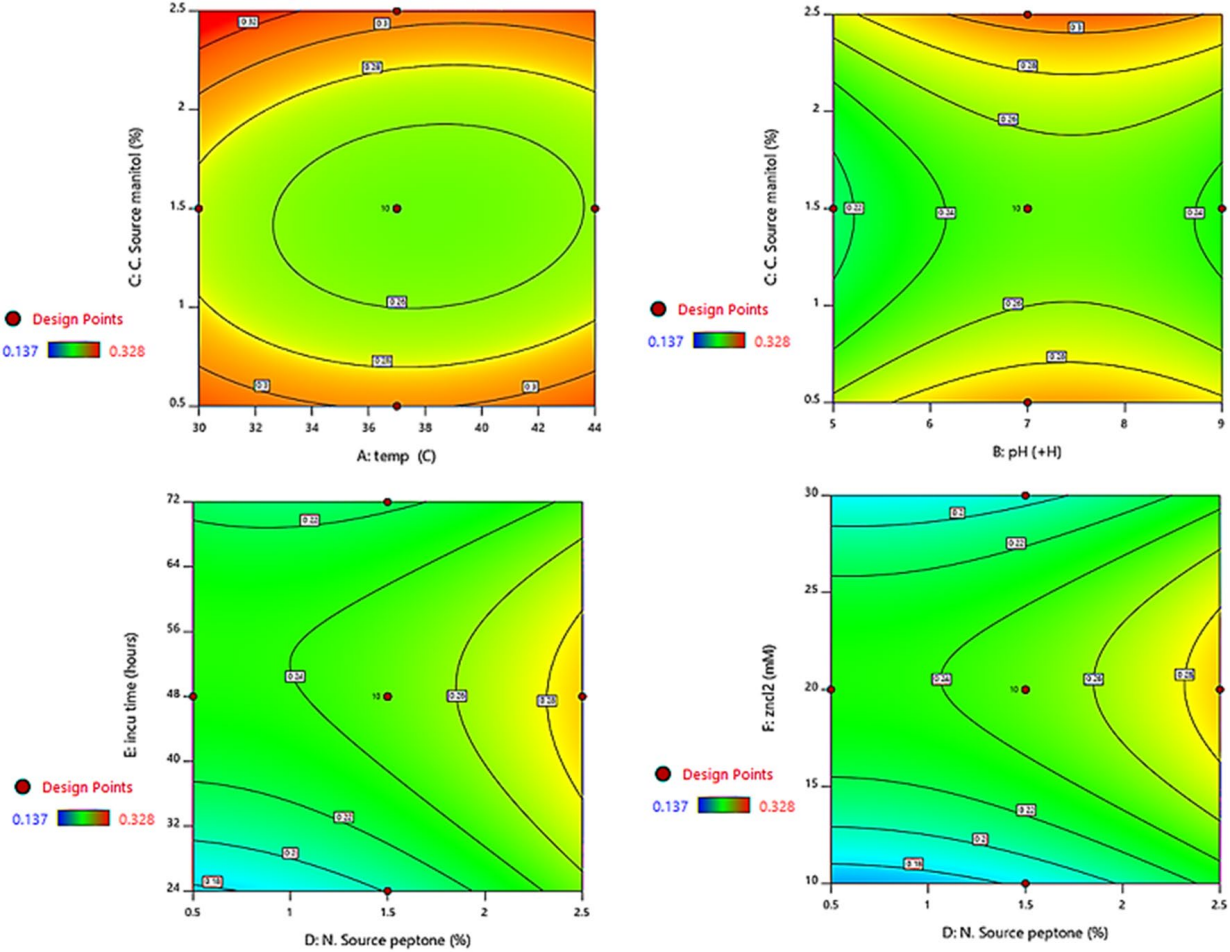
Contour plots of RSM optimization of STX production showing the interaction between two independent nutritional variables. Contour lines and bands show the response values (STX amonut) based on the different combinations of the variables. The plots used different colors to depict different levels of STX production between two independent parameters while holding the third constant. The graphs show that each parameter has a considerable impact on STX production

RSM optimization using a central composite design was implemented in our previous report [9] to evaluate the fundamental and interaction effects of different fermentation factors on the synthesis of STX. According to the data, the most significant improvement in STX pigmentation from *S. aureus* A2 strain occurred under particular culture circumstances. These conditions included a concentration of peptone of 1.5%, a concentration of mannitol of 2.5%, an incubation period of 48 h, a temperature of 37 °C, a pH of 5, and a concentration of ZnCl2 of 20 mM. The interaction effects of the examined factors in optimizing the output of STX between any two independent variables were depicted through contour graphs, as shown in Fig. 6. The usage of RSM is becoming increasingly popular due to its capacity to collect ideal settings for multi-factorial studies in an effective and efficient manner. The findings of this study are in line with those of previous research that investigated the application of RSM to enhance the conditions of pigment production cultures. They used RSM to fine-tune the growing conditions for the highly prolific strain of *Micrococcus luteus* (ATCC 9341) in order to maximize the production of carotenoid. This was done in order to optimize carotenoid output. At a temperature of 32.5 °C, the culture conditions were adjusted to include 3% whey, 175 rpm of agitation, pH 7, and 7.5% inoculum size [74]. RSM was also implemented by El-Zawawy et al. to cultivate Streptomyces djakartensis NSS-3 on low-cost agricultural waste for optimizing the production of melanin [75]. With a peak extraction time of 21.14 min, a temperature of 52.98 °C, and a solid-to-liquid ratio of 21.61 mg/mL, Prabhu et al. utilized RSM to improve and optimize the extraction of Betalain pigments from the promising Beta vulgaris [76]. RSM shows enhanced precision over conventional techniques in maximizing pigment production.

### Extraction process of staphyloxanthin

The microbial production of carotenoids is intracellular. In line with other intracellular bioproducts, a series of downstream operation units are employed for the recovery and processing of carotenoids after the fermentation phase. The cellular biomass, which contains intracellular carotenoids, is typically segregated from the supernatant during the initial clarification stage, which involves the use of conventional filteration or centrifugation operations [77]. The process of carotenoid extraction from microbial detritus consists of two stages: the disruption of the microbial cell membrane and the subsequent extraction of carotenoids [78]. For the recovery of microbial products from other metabolites and cell lysate, solvent extraction provides a high extraction efficiency of carotenoids. The utilization of methanol for solvent extraction is regarded as the standard approach for isolating STX pigment from *S. aureus* [79]. Given that exposure to high temperatures, or light can readily degrade carotenoids. It is imperative to select the appropriate steps and procedures in order to preserve their stability [80]. Therefore, solvent extraction process of STX pigment is performed in dark environment at low temperature or ambient conditions.

**Fig. 7:**
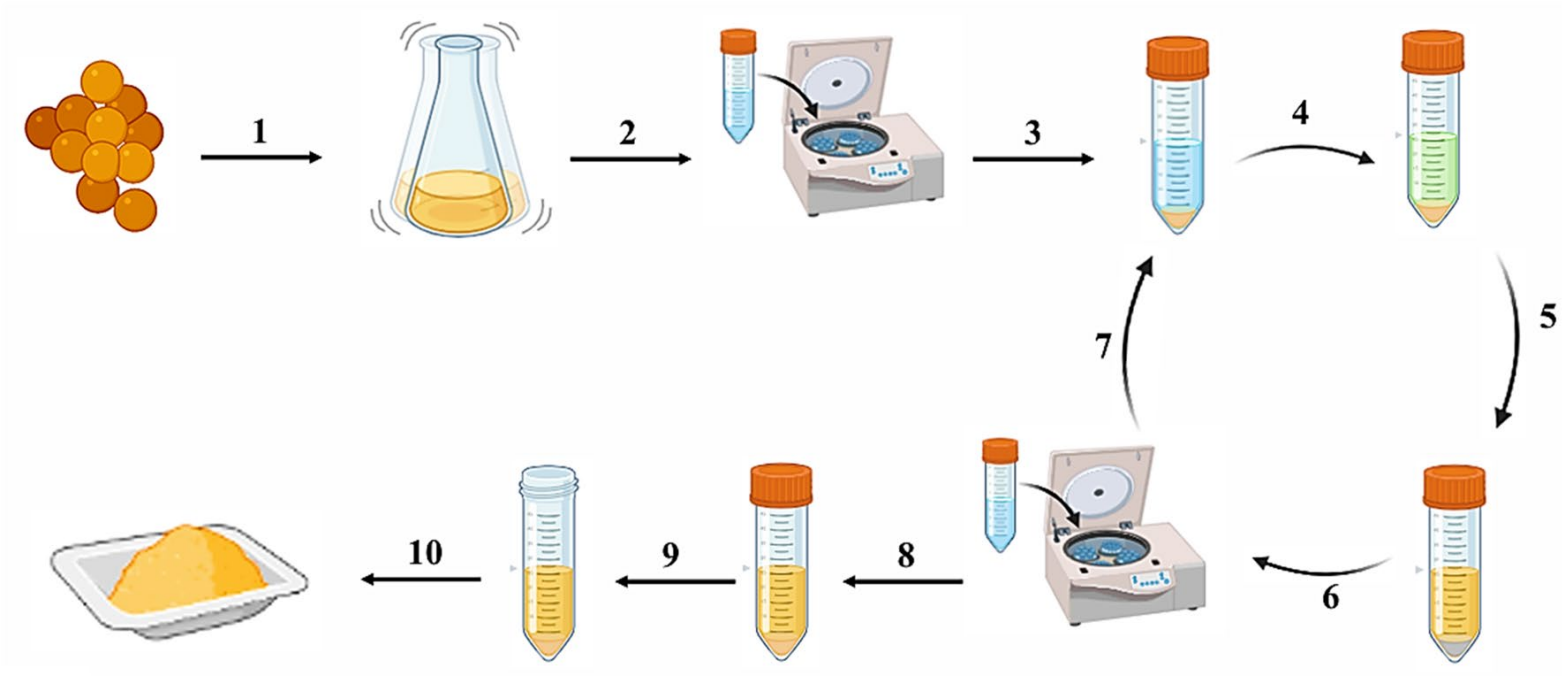
Schematic diagram illustrated the processes involved in the production and extraction of the golden yellow STX pigment derived from *S. aureus*. Several steps involved in the extraction process can be summarized as follows: (1) Incubation of the pigmented bacteria for enhancement of STX production followed by centrifugation and washing of cell pellets (steps 1–3). (2) Solvent extraction through overnight incubation in the dark in methanol until the pellets were bleached, followed by centrifugation (steps 4–7). (3) The pigmented supernatant underwent a process of solvent evaporation, yielding a dry orange powder (steps 8–10)

In this section, the extraction stages of STX were illustrated in accordance with the previous reports [9, 79, 81–84] to simplify and summarize the process of obtaining a high yield of STX, as shown in Fig. 7. Step 1 involved the incubation of highly pigmented *S. aureus* in the selected medium for STX production and incubation at optimal conditions. The medium’s color changed to a golden yellow pigmentation as a result of the generation of STX pigment (Step 2). The culture medium underwent centrifugation at 8000 x g for 10 min to facilitate the separation of highly pigmented bacterial cells. Following centrifugation, the resulting cell pellets were resuspended in phosphate buffered saline (PBS) to facilitate washing and the removal of residual culture medium (Step 3). The procedure was conducted three times to guarantee the clarity of the resulting supernatant after centrifugation. The resulting pigmented cell pellets were mixed with absolute methanol in a 4:1 (solvents/pellets, v/w) ratio to start the pigment extraction process. The mixture was then resuspended by vortexing and pipetting until all of the clumps were disintegrated (step 4). The reaction mixture underwent incubation in a dark environment overnight to ensure thorough extraction (step 5), after which the supernatants containing the extracted pigment were collected through centrifugation at 10,000 x g at 4 °C for 15 min (step 6). The pellets underwent a complete extraction process with solvent on several times until they exhibited a complete loss of color (step 7). Upon the thorough extraction and subsequent centrifugation, the clarified supernatants were transferred to a dry and sterile tube (step 8), where they underwent a process of solvent evaporation in a controlled dark environment (step 9), ultimately yielding a dry orange powder. The resultant orange STX powder (Step 10) is suitable for storage at − 18 °C for subsequent procedures.

### Analytical techniques for purification and characterization of staphyloxanthin

Various analytical procedures can be employed to separate the microbial carotenoids that are recovered following the extraction stage. There are numerous analytical techniques that have been implemented to facilitate the purification of carotenoids [78]. Separation methods are chosen in accordance with the physical and chemical characteristics of the biosubstances that require separation. The following approaches of separation are frequently employed in the separation of biosubstance mixtures [85]: Distillation is a method that involves the selective evaporation and condensation of liquid mixtures to separate their constituent substances; The process of solid crystals forming from a melt, solution, or, less frequently, directly from a gas is known as crystallization; Dialysis employs a semipermeable membrane, such as dialysis tubing, to distinguish molecules in solution according to variations in their dialysis rates; The process of electrophoresis separates organic molecules according to their diametrically opposed interactions with a gel under an electric potential, or diametrically opposed movement, and is used to separate and analyze macromolecules and their fragments according to their size and charge; A physical separation method known as column chromatography and thin layer chromatography involves the distribution of components between two stationary phases, the stationary phase and the mobile phase, which are moving in a specific direction.

**Fig. 8:**
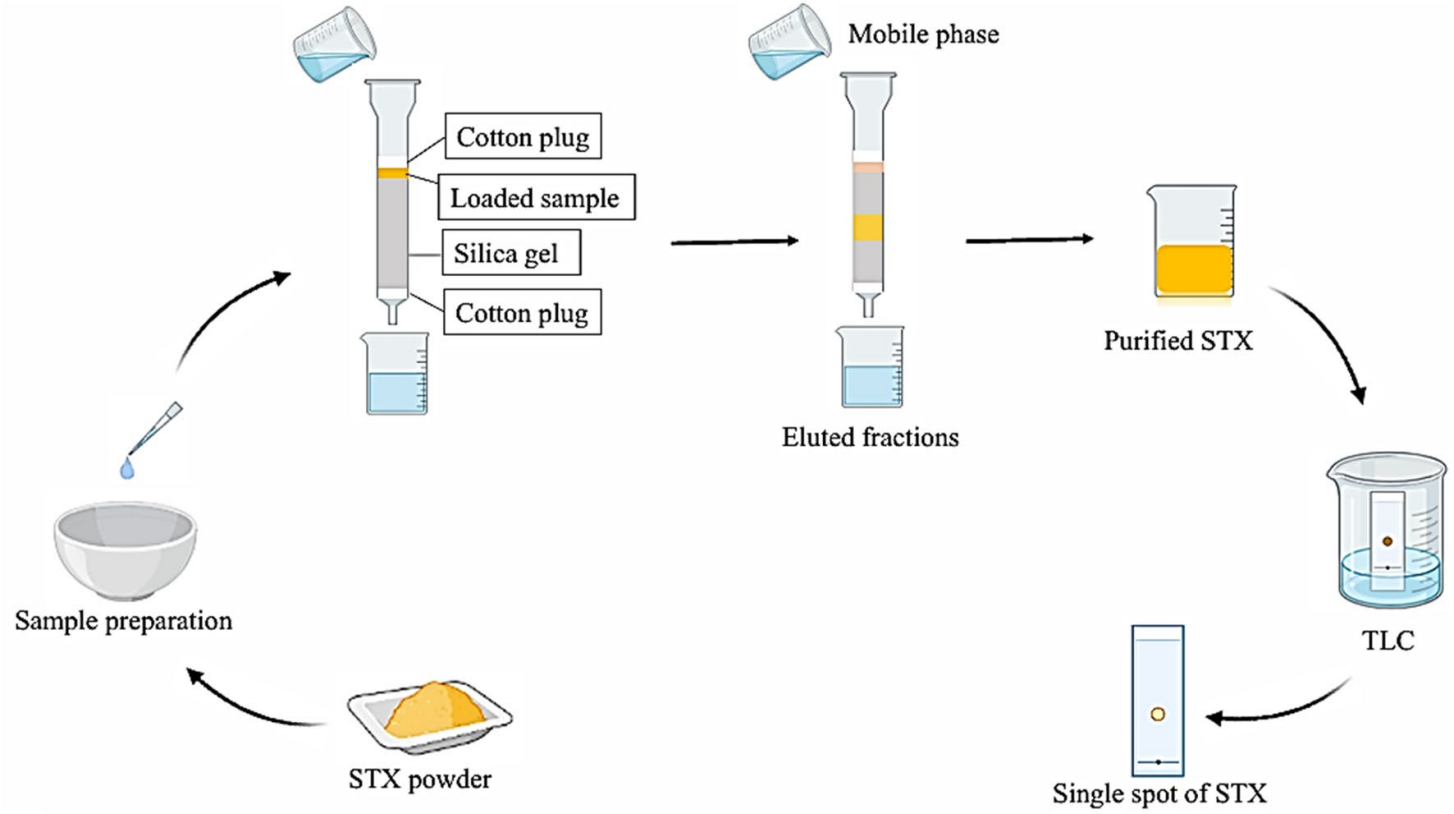
Schematic diagram illustrating the purification of the extracted STX pigment. The sample is prepared and loaded onto the surface of the silica gel column chromatogram and eluted using fractionated mobile phase for separation of impurities. The purity of STX pigment is checked through the appearance of single spot on thin layer chromatography plate

Column chromatography and thin layer chromatography (TLC) are established methods utilized for the purification of STX, according to the literature [9, 82, 86, 87]. This section illustrates the purification stages of STX, aligning with previous reports to streamline and summarize the process of obtaining purified STX, as depicted in Fig. 8. The initial step involves the preparation of the extracted STX for the packing of the column chromatogram. The dried orange STX powder is dissolved in a minimal volume of methanol and combined with a portion of used silica gel powder to enhance the loading of the STX pigment onto the surface of the fine silica gel particles. To remove the impurities, the sample was defatted by eluting it with n-hexane and then with 100% chloroform after the column chromatogram was packed. The polarity of the mobile phase was enhanced through the incorporation of ethanol, beginning with a chloroform: ethanol mixture at a ratio of 99:1 (v/v). A satisfactory elution of the sample was achieved by employing the fractionated mobile phase until the appearance of the distinctive golden-yellow band, as illustrated in Fig. 8. The eluted fractions, characterized by a golden yellow color, were collected and subsequently subjected to evaporation of the mobile phase on a porcelain plate at room temperature and in darkness. The purity and identity of STX is checked using TLC, as shown in Fig. 8. On the activated TLC plate, a thin line was traced approximately 1.5 cm above the bottom. An area of the extract was placed on the line and allowed to dry. Succeeding this, the extract was reintroduced at the same location. The plate was subsequently immersed in a container that was entirely saturated with a methylene chloride: ethanol mixture at a ratio of 9.5: 0.5 (v/v). The solvents were permitted to ascend on the plate until they reached a height of 1.5 cm, where they were in close proximity to the summit. Following its removal, the retardation factor (R_f_) was determined. The appearance of single spot on a TLC plate with a R_f_ of 0.38 indicates the purity and identity of STX [9, 84, 88].

A variety of analytical techniques have been employed for the characterization and identification of STX, a process that may be influenced by its biosynthetic intermediates, as outlined in Table 1 [9, 15, 82, 88]. It was determined that the purified STX had a maximum absorption wavelength of 456 nm throughout the UV–vis spectrum. The identification of the functional groups of the molecule can be facilitated through the use of Fourier transform infrared spectroscopy (FTIR). Based on the research conducted by Nosair et al. and Barretto & Vootla, it was found that the FTIR absorption spectra revealed the presence of OH groups at 3390 cm-1 and C = O groups at 1648 cm-1. The 2924 cm − 1 peaks were determined to be the aliphatic CH stretch, whereas the 1110 –1024 cm-1 peaks were determined to be the ester group stretch. There was also the observation of a C-C stretch at 1456 cm-1. The authors suggested that the functional groups observed are a consequence of the presence of specific components in the STX structure, including fatty acid ester, methyltetracosane, and β–D-glucopyranose [9, 82]. The most frequently employed method for the identification and characterization of carotenoids is mass spectrometry (MS). In the presence of the precursor ion [M + H]+, the molecular mass of STX was 819.33 m/z. In addition, similar to the fragmentation pattern observed in the published data, the MS/MS results validated the molecular structure by revealing fragment ions at 415.1 and 431.1 m/z [15, 82].

Nevertheless, it is crucial to recognize that stereoisomers cannot be distinguished by MS methods. Therefore, methods based on nuclear magnetic resonance (NMR) spectroscopy have to be implemented. It is confirmed by the NMR structure elucidation that the pigment is STX, which is consistent with previous reports. According to Pelz et al. [15], STX was determined to be a β-D-glucopyranosyl 1-O-(4,4-diaponeurosporen-4 oate)-6-O-(12-methyltetradecanoate) by analysis of the NMR spectra. At position C6 with the C15 fatty acid 12-methyl tetradecanoic acid and the glucose esters at position C1 with the carotenoids 4,4-diaponeurosporenic acid form the structure’s center core. According to Barretto and Vootla [82], the CDCl_3_ solvent was used to acquire the ^1^H spectra of the extracted STX pigment. While C25-H, C21-H, and C17-H reverberated at 7.28, 6.45, and 3.65 ppm, respectively, and the protons were in the predicted zone, researchers discovered that the The-OH protons of C20, C19, and C18 resonated at 4.38, 4.83, and 4.97 ppm, respectively.

NIR-FT-Raman spectroscopy is another cutting-edge technique for defining carotenoids. It is typically thought of as a quick and non-destructive method that allows these biomolecules to be characterized based on the distinctive bands of the most prevalent carotenoids. NIR-FT-Raman spectroscopy is a very essential tool for the routine characterization of carotenoids because it is a reasonably easy and non-destructive approach [89]. Siems et al. [90] identified the STX pigment produced by colonies of four *Staphylococcus capitis* subsp. capitis strains using normalized average Raman spectra. The authors observed two distinct peaks at 1525 cm − 1 and 1160 cm − 1 in the Raman spectra of STX. The peaks represent the –C = C- and = C–C = bonds of STX pigment. Label-Free Raman Spectroscopy was employed by Pistiki et al. [91] to differentiate different *S. aureus* isolates. The dominant STX bands were the basis for the authors’ determination of the strains’ differences. At 1523, 1160, and 1007 cm, the most noticeable peaks in the difference spectrum, it is possible to discern the typical spectral pattern of carotenoids in the mean spectrum. STX, a carotenoid and a distinctive golden pigment of *S. aureus* found in most strains, is the primary source of these signals.

It is of the utmost significance to characterize carotenoids; however, the accurate quantification of the number of carotenoids during the production and extraction processing stages is more critical, as it determines the overall yields of the process. The most precise analytical method for measuring and identifying microbial carotenoids is high-performance liquid chromatography (HPLC); nonetheless, the operation’s success is directly influenced by processual factors, including stationary and mobile phases, detector type, and others [92]. The solvents water, acetone, methanol, dichloromethane, isopropanol, and acetonitrile, which can be employed as pure or mixed solutions, are frequently utilized for the HPLC quantification of carotenoids. For instance, STX can be eluted with a retention time of 0.645 when acetonitrile : methanol at a 75:25 is employed as a mobile phase [82]. A further study examined the identification of STX utilizing an HPLC-DAD system in conjunction with a coupled mass spectrometer (HPLC-DAD-APCI-MSn) [90]. The STX was eluted with a retention time peak at 10.02 min using the solvent system of methanol (MS grade) : methyl tert-butyl ether (MTBE: ≥99.0%) : H_2_O (85:5:10, v/v/v).

**Table 1:** Characterization of Staphyloxanthin and its biosynthetic intermediates

Clones	Produced products	Maximum absorption (λ max)	FTIR peaks (functional groups)	Mass analysis m/z [M + H]^+^	References
*crtM*	Dehydrosqualene	287	CH stretch, C-C stretch	-	[93]
*crtMN*	4,4’-Diaponeurosporene	415,438,468	CH stretch, C-C stretch	-	[15, 88]
*aldH*	4,4’-Diaponeurosporen-4-al	466	C = O group, CH stretch, C-C stretch	415.1	[15, 88]
*crtQMN*	4,4’-diaponeurosporenoate	455	C = O, OH groups, CH stretch, C-C stretch	433.1	[15, 88]
*crtPQMN*	Glycosyl-4,4’-diaponeurosporenoate	460,483	C = O, OH groups, CH stretch, C-C stretch, C–O stretch	594.7	[15, 88]
*crtOPQMN*	Staphyoxanthin	456	C = O, OH groups, CH stretch, C-C stretch, C–O stretch	819.3	[9, 15, 81, 82, 88]

### Biotechnological applications of staphyloxanthin

**Fig. 9:**
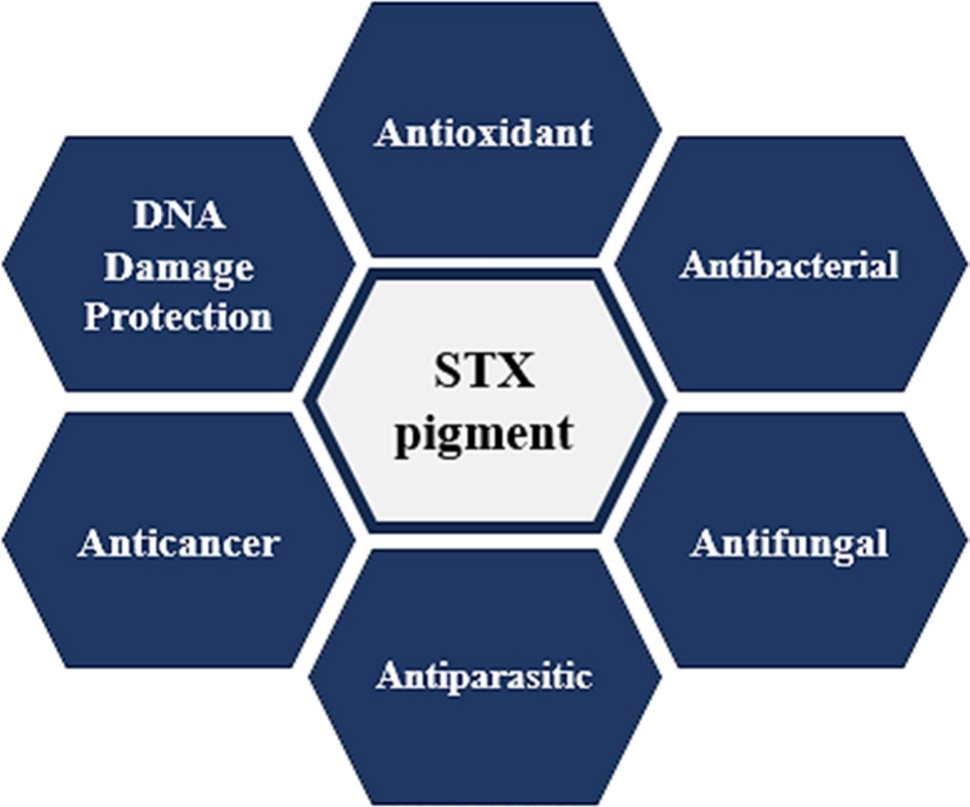
Biotechnological applications of staphyloxanthin in healthcare field

Pigments, which are generated as secondary metabolites to safeguard microorganisms from detrimental impact, also possess numerous biological applications. The applications of STX have been examined in this review, and it has been demonstrated that it has the potential to be used as a prospective agent in healthcare field (Fig. 9). Nevertheless, continued research is indeed required to elucidate its novel functions.

### Staphyloxanthin as a anti-oxidant agent

The cell generates reactive oxygen species (ROS) and reactive nitrogen species (RNS) during aerobic metabolism processes, which encompass the activation of cell signaling cascades, gene expression, and signal transduction. The integrity of cell membrane structures, gene expression, and enzyme functions can be adversely affected by ROS, which can damage biologically important molecules such as proteins, DNA, and lipids. It is widely recognized that ROS are implicated in the patho-biochemistry of degenerative diseases [94]. An antioxidant is defined as any substance that inhibits, prevents, or mitigates oxidative damage to a target molecule. Several enzymatic and non-enzymatic antioxidants are incorporated into the intricate networks of antioxidant defense systems in living organisms. Carotenoids are recognized for their significant contributions to the scavenging of reactive oxygen species (ROS), including peroxyl radicals and singlet molecular oxygen (^1^O2). However, there is a lack of information regarding their involvement in cellular defenses against RNS [95]. Our carotenoid of interest, STX, has been documented as a potent anti-oxidant agent. Based on the results of a singlet oxygen experiment and predictions made by fluorescence-based ROS quantification, Valliammai et al. [81] showed that the STX molecule scavenges free radicals with its conjugated double bonds. Another study found that, in comparison to normal ascorbic acid, which has an IC_50_ value of 35.54 µg/mL, STX exhibited significant 1, 1-diphenyl 2-picrylhydrazyl (DPPH) free radical scavenging activity (%) with an IC_50_ value of 54.22 µg/mL [82].

### Staphyloxanthin with DNA damage protection activity

The significant genetic characteristics of living organisms are regulated by deoxyribonucleic acid (DNA), a complex macromolecule. Important DNA segments known as genes play an indirect role in the coding of proteins, which serve as the fundamental building blocks of biological systems. Certain forms of DNA, their structures, and the roles that they perform within the human body are responsible for the majority of the genetic information, abnormalities, and diseases that are studied [96]. The functionality of DNA within cells is influenced by a variety of factors, including environmental factors, synthetic chemicals, UV radiation, and genetic defects, which ultimately leads to considerable alterations in living organisms [97]. Extracts derived from various natural resources may help prevent or slow down DNA damage. This will contribute to the elimination of severe DNA-related diseases in humanity [98]. Pelz et al. reported that the STX pigment demonstrated notable protective activity against DNA damage. During the reaction with Fenton’s reagent, which produces ROS, the pBR322 plasmid DNA undergoes cleavage, resulting in DNA stress and damage. Compared to the supercoiled and open circular bands of the pBR322 plasmid DNA control, the STX-treated DNA was observed to be intact on the agarose gel picture [82]. Pannu et al. [21] demonstrated that STX improved the DNA damage repair mechanism in *S. aureus* following UV and X-ray irradiation.

### Staphyloxanthin as an anticancer agent

The uncontrolled proliferation of abnormal cells within the body is the definitive characteristic of cancer. There is an irreversible initial stage of cancer that results in an increase in the proliferation of tumor cells. Global Cancer Statistics (GLOBOCAN) indicates that cancer incidence and mortality are increasing at an accelerated pace on a global scale [99]. The development of cancer can be influenced by the carcinogenic factors, which are characterized by hyperplastic, dysplastic, or regenerative alterations that disrupt cellular homeostasis [100]. The anti-cancer properties of carotenoids have been demonstrated in a multitude of in vitro and in vivo studies. Carotenoids may help prevent prostate, oral, lung, leukemic, intestinal, hepatic, breast, and bladder cancers, according to the findings of these research. Carotenoids have anti-cancer properties through a number of ways, such as inhibiting cell proliferation and inducing cell apoptosis [95, 101]. The primary mechanism by which carotenoids exert their preventive anticancer effects is through their potent antioxidant and pro-oxidants properties, which may mitigate DNA damage caused by ROS [102]. Under situations that are characterized by high concentrations, high oxygen tension, and an imbalanced cellular redox state, carotenoids, on the other hand, operate as pro-oxidant agents. Oxidative stress, which impedes cancer progression and metastasis, is induced by the pro-oxidative properties of carotenoids, which contribute to the production of ROS in cancer cells. Apoptosis is promoted by pro-oxidants that enhance ROS signaling pathways and/or weaken the antioxidant defenses of cancer cells [103].

Numerous studies have examined the capacity of our carotenoid of interest, STX, to affect a variety of cancer types. Hassan et al. [87] reported the cytotoxic efficacy of STX against breast cancer. The study demonstrated the significant efficacy of STX in inhibiting the growth of the MCF-7 cancer cell line, with enhanced cytotoxicity observed at higher pigment concentrations and extended exposure durations. An additional study examined the potential of the STX pigment as an anticancer agent that is biocompatible and capable of destroying cancer cells at extremely low concentrations without causing damage to normal cells [82]. The authors assessed the cytotoxic effects and cell antiproliferative activity of STX pigment on four established cancer cell lines: Mus mucus skin melanoma (B16F10), Adenocarcinomic human alveolar basal epithelial cells (A549 lung carcinoma), Dalton’s lymphoma ascites (DLA), and Ehrlich ascites carcinoma (EAC). According to Algabar et al. [104], STX has the potential to impact lung cancer cells (A549), hepatocellular carcinoma HepG2 cells, and breast adenocarcinoma (MCF7). This effect is mediated by acute ROS production, DNA damage, and subsequent oxidative stress, and cellular toxicity. Additionaly, Nosair et al. [9] reported the cytotoxic efficacy of STX produced from *S. aureus* on non-smal cell lun cancer (NSCLC). In this study, STX demonstrated a disrupted cell cycle at the G0/G1 and pre-G1 phases in the promotion of apoptosis in A549 cancer. The percentage of the necrotic cells increased from 0.16% to 0.22%, the late apoptotic cells improved from 1.97% to 4.47%, and early apoptotic cells increased from 1.6% to 15.82%. This research indicated that STX were predominantly initiated by the apoptotic pathway, instead of the necrotic effect. Based on the fact that the inhibition of the epidermal growth factor receptor (EGFR) is a contemporary approach to combating cancer proliferation, and the EGFR plays a vital part in intracellular signaling, differentiation, and morphogenesis [105, 106]. The authors found that reducing EGFR expression by utilizing potential binding modalities with both the mutant and wild-type EGFR active sites demonstrated the antitumor activity of STX.

### Staphyloxanthin as an antimicrobial agent

The potential applications of carotenoids in combating bacterial and fungal infections are attributed to their ability to generate ROS. This process induces oxidative stress, disrupts ion-membrane interactions, and modifies cellular permeability [103, 107]. Antibacterial and antifungal activity of STX against both gram-positive and gram-negative bacteria has been discussed by numerous investigations, as sumarized in Table 2. Barretto and Vootla [82] investigated the antimicrobial properties of STX in relation to gram-negative bacteria *E. coli* and yeast *Candida albicans*. *E. coli* achieved the highest level of inhibition, with a minimum inhibitory concentration (MIC) of 25 µg/mL, followed by *Candida albicans* with a MIC value of 50 µg/mL. The investigation carried out by Algabar et al. [108] revealed that STX demonstrated antibacterial properties against various pathogenic bacteria, including *Serratia marcescens*, *Klebsiella pneumoniae*, *Pseudomonas aeruginosa*, and *Enterobacter* species, with the most significant activity noted against P. aeruginosa, which exhibited an inhibition zone of 15 mm. On the other hand, AL-Kazaz et al. [84] revealed that STX showed no antibacterial activity against all the tested bacteria (*Pseudomonas putida*, *Pseudomonas fluorescens*, *Proteus* spp, *Klebsiella* spp, *E. coli*, *Shigella* spp, *P. aeruginosa*, *Salmonella* spp).

**Table 2:** Antimicrobial activity of Staphyloxanthin extracted from *S. aureus*

Authors	Microorganisms	Methods	STX concentration
Barretto and Vootla [82]	*Escherichia coli*	Agar well diffusion method	Different concentrations (100, 75, 50, and 25 µg/mL in methanol)
	*Candida albicans*		
AL-Kazaz et al. [84]	*Pseudomonas aeruginosa*	Agar well diffusion method	0.2 g/mL
	*Pseudomonas putidae*		
	*Pseudomonas fluorescence*		
	*Escherichia coli*		
	*Klebsiella spp.*		
	*Salmonella spp.*		
	*Shigella spp.*		
	*Proteus spp.*		
Algabar et al. [108]	*Pseudomonas aeruginosa*	Agar well diffusion method and turbidity method	Two concentrations of 2 and 20 mg/mL
	*Serratia marcescen*		
	*Klebsiella pneumoniae*		
	*Enterobacter spp.*		

### Staphyloxanthin as an antiparasitic agent

**Fig. 10:**
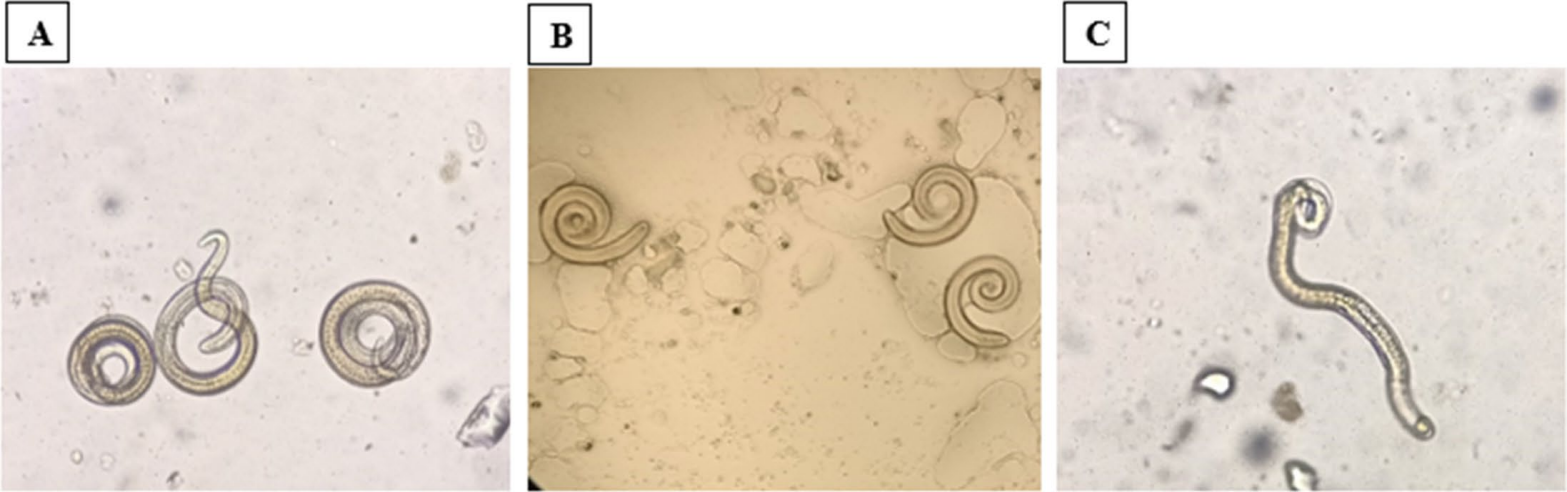
Antiparasitic activity of STX against *Trichinella spiralis* showing the viability and mortality of muscle larvae.** A** viable muscle larvae showing coiling movements.** B** comma-shaped immotile muscle larvae.** C** straight immotile muscle larvae

Anthelmintic agents can cause paralysis and consequent mortality by damaging the cuticle or cytoskeleton of parasites or impairing their metabolism [109]. The antiparasitic capability of carotenoids is predominantly attributed to the production of reactive oxygen species, which in turn results in oxidative stress and changes in cell permeability [103]. Garcia-Bustos et al. speculated that the pro-oxidative traits increase the anthelmintic potential of helminth parasites by attachment to the glycoprotein on their cuticle, and disrupting their decoupling oxidative phosphorylation and energy generation, resulting in their mortality [110]. Additionally, Coronel et al. have shown that carotenoids have the potential to decouple specific reductase-mediated mechanisms that constrain the energy production capabilities of helminth parasites [111]. The chemical nature of carotenoids is the primary factor contributing to the antiparasitic activity of STX. Our work on the effect of STX produced by *S. aureus* against *Trichinella spiralis* infection showed diminished larval viability, as the presence of immotile, comma-shaped, or straight larvae is a characteristic indicator of mortality (Fig. 10), associated with membrane blebbing, numerous notches, and excessive cuticular deformities [112]. Moreover, the oral administration of STX-encapsulated niosomes exhibited improved therapeutic efficacy in mice relative to the reference drug, albendazole. The eradication of adult worms, improvement in histopathological characteristics, reduction in larval numbers, and significant decrease in inflammatory expression around trichina capsules all indicated this result.

## Conclusion and future perspectives

*Staphylococcus aureus* serves as a microbial cell factory for the production of staphyloxanthin, providing high pigment yield. This review addresses advances in STX synthesis and bioproduction in *S. aureus*, focusing on the biosynthetic pathway and optimization strategies, which offer significant potential for enhancing STX production. The extraction process, purification, and analytical characterization have significantly improved the application of purified STX in biomedical contexts, serving as an antibacterial, antifungal, antiparasitic, antioxidant, anticancer, and DNA damage protection agent.

Future research should explore additional optimization trials by incorporating factors such as oxidative stress and agro-industrial by-products to enhance STX production. Further exploration of the utilization of integrated upstream and downstream platforms, as well as the development of innovative and low-energy extraction techniques, may enhance the extraction yields and revenues. The integration of various encapsulation techniques, such as encapsulation with supercritical fluids, nanoencapsulation, or microencapsulation, could be employed to enhance the stability of STX. In addition to chemotherapeutic agents, STX may be employed as an adjuvant agent in the treatment protocol of cancer therapy to enhance the efficacy of these agents and reduce the possibility of resistance. In order to find novel therapeutic properties of STX pigment, future bioprospecting is necessary, with the goal of developing environmentally friendly applications in a variety of other disciplines.

## Data Availability

No datasets were generated or analysed during the current study.
